# Effect of *κ*-opioid receptor agonist on the growth of non-small cell lung cancer (NSCLC) cells

**DOI:** 10.1038/bjc.2011.574

**Published:** 2012-02-16

**Authors:** N Kuzumaki, A Suzuki, M Narita, T Hosoya, A Nagasawa, S Imai, K Yamamizu, H Morita, H Nagase, Y Okada, H J Okano, J K Yamashita, H Okano, T Suzuki, M Narita

**Affiliations:** 1 Department of Physiology, Keio University, School of Medicine, 35 Shinanomachi Shinjuku-ku, Tokyo 160-8582, Japan; 2 Department of Pharmacology, Hoshi University School of Pharmacy and Pharmaceutical Sciences, 2-4-41 Ebara, Shinagawa-ku, Tokyo 142-8501, Japan; 3 Department of Toxicology, Hoshi University School of Pharmacy and Pharmaceutical Sciences, 2-4-41 Ebara, Shinagawa-ku, Tokyo 142-8501, Japan; 4 Biological Systems Control Team Biomedicinal Information Research Center, Aomi 2-4-7, Koto-ku, Tokyo 135-0064, Japan; 5 Laboratory of Stem Cell Differentiation, Stem Cell Research Center, Institute for Frontier Medical Sciences, Kyoto University, 53 Shogoin Kawahara-cho, Sakyo-ku, Kyoto 606-8507, Japan; 6 Faculty of Pharmaceutical Sciences, Hoshi University School of Pharmacy and Pharmaceutical Sciences, 2-4-41 Ebara, Shinagawa-ku, Tokyo 142-8501, Japan; 7 Laboratory of Medical Chemistry, School of Pharmacy, Kitasato University, Tokyo, Japan

**Keywords:** *κ*-opioid receptor, non-small cell lung cancer, gefitinib

## Abstract

**Background:**

It is becoming increasingly recognised that opioids are responsible for tumour growth. However, the effects of opioids on tumour growth have been controversial.

**Methods:**

The effects of *κ*-opioid receptor (KOR) agonist on the growth of non-small cell lung cancer (NSCLC) cells were assessed by a cell proliferation assay. Western blotting was performed to ascertain the mechanism by which treatment with KOR agonist suppresses tumour growth.

**Results:**

Addition of the selective KOR agonist U50,488H to gefitinib-sensitive (HCC827) and gefitinib-resistant (H1975) NSCLC cells produced a concentration-dependent decrease in their growth. These effects were abolished by co-treatment with the selective KOR antagonist nor-BNI. Furthermore, the growth-inhibitory effect of gefitinib in HCC827 cells was further enhanced by co-treatment with U50,488H. With regard to the inhibition of tumour growth, the addition of U50, 488H to H1975 cells produced a concentration-dependent decrease in phosphorylated-glycogen synthase kinase 3*β* (p-GSK3*β*).

**Conclusion:**

The present results showed that stimulation of KOR reduces the growth of gefitinib-resistant NSCLC cells through the activation of GSK3*β*.

Opioids are small endogenously produced peptide molecules that are widely known for their analgesic and psychoactive properties (Ciccone *et al*, 1980; Zubieta *et al*, 2001; Moles *et al*, 2004). It has been shown that opioids can promote the growth of tumour cells (Lazarczyk *et al*, 2010). On the other hand, it has been controversially reported that opioids induce the apoptosis of immunocytes, cancer cells and neuroblastoma cells (Boehncke *et al*, 2010). Thus, it is becoming increasingly recognised that opioids have a role in tumour growth (Saurer *et al*, 2008).

Three major types of opioid receptors, *μ*, *δ* and *κ*, have been well characterised. *κ*-Opioid receptors (KORs) are widely expressed throughout the central nervous system (Chavkin *et al*, 1982; Dhawan *et al*, 1996). It has been reported that KOR is also expressed in the human adenocarcinoma breast cancer cell line MCF7 and small cell lung carcinoma (Kallergi *et al*, 2003). Furthermore, KOR agonist has been shown to inhibit the growth of H157 cell, which is a non-small cell lung cancer (NSCLC) cell (Maneckjee and Minna, 1990). However, little is known about the mechanism that underlies the inhibitory effect of KOR stimulation on the growth of NSCLC cells.

Epidermal growth factor receptor (EGFR) is a major target of molecular anti-NSCLC therapy (Wakeling *et al*, 2002). Non-small cell lung cancer patients with L858R or exon 19 deletion mutations in EGFR show good responses to the tyrosine kinase inhibitor gefitinib. However, patients with wild-type EGFR and acquired mutation in EGFR T790M are eventually resistant to treatment with gefitinib. In this study, we examined whether the selective KOR agonist U50,488H could inhibit the growth of gefitinib-sensitive and EGFR mutant (delE746-A750, L858R) NSCLC cells (HCC827) and gefitinib-resistant and EGFR mutant (T790M) NSCLC cells (H1975), and investigated the signalling mechanism of the KOR-mediated inhibitory effect on tumour cell growth.

## Materials and methods

### Cell culture

The human NSCLC cell lines HCC827 and NCI-H1975 (H1975; both from American Type Culture Collection Co., MD, USA) were cultured in HEPES-modified RPMI 1640 medium (Sigma-Aldrich Co., St Louis, MO, USA) with 10% fetal bovine serum (FBS; Invitrogen Life Technologies Co., Carlsbad, CA, USA) and 1% penicillin-streptomycin (Invitrogen Life Technologies Co.). Normal human lung fibroblasts (NHLF; Lonza Inc., Allendale, NJ, USA) were cultured in fibroblast basal medium with insulin, rhFGF-B, GA-1000 and FBS (all from Takara Bio Inc., Tokyo, Japan). All cells were maintained under a humidified atmosphere of 5% CO_2_ at 37°C.

### Reagents

The reagents used in the present study were gefitinib (Toronto Research Chemicals Inc., Canada), (±) trans 3,4-dichloro-*N*-methyl-*N*-(2-(1-pyrrolidinyl) cyclohexyl)-benzeneacetamide (U50,488H) methanesulfonate (Sigma Chemical Co.), nor-binaltorphimine dihydrochloride (nor-BNI; Tocris Cookson Ltd., St Louis, MO, USA), and 6-bromoindirubin-3′-oxime (BIO; WAKO Pure Chemical Industries Ltd., Osaka, Japan).

### Cell viability assay

Cell viability was determined by a cell proliferation assay using 3-(4,5-dimethylthiazol-2-yl)-2,5-diphenyltetrazolium bromide, yellow tetrazole (MTT). A 20-*μ*l of MTT solution (5 mg ml^−1^) was added to each well of the culture medium. After incubation for an additional 2 h, the medium was removed and 100 *μ*l of DMSO was added to resolve the formazan crystals. Optical density was measured using a microplate reader with an absorption wavelength of 600 nm. In each experiment, three replicates were prepared for each sample. The proportion of living cells was determined based on the difference in absorbance between the samples and controls.

### Immunohistochemistry

The procedure for immunohistochemistry is described in the Supplementary Methods.

### RNA preparation and semiquantitative analysis by reverse transcription (RT)-PCR

The RNA preparation and RT-PCR method are described in the Supplementary Methods.

### Western blotting

Sample preparation and loading for western blotting are described in the Supplementary Methods. For immunoblot detection, membranes were blocked in Tris-buffered saline (TBS) containing 1% non-fat milk (Bio-Rad Laboratories, Hercules, CA, USA) containing 0.1% Tween 20 (Sigma-Aldrich Co.) for 1 h at room temperature with agitation. The membrane was incubated with primary antibody diluted in TBS (1 : 1000 phosphorylated-EGFR (Cell Signaling Technology Inc., Boston, MA, USA), 1 : 500 p-Akt (Cell Signaling Technology Inc.), 1 : 1000 p-GSK3*β* (Cell Signaling Technology Inc.), 1 : 2000 p-STAT3 (Cell Signaling Technology Inc.), 1 : 750 GSK3*β* (Santa Cruz Biotechnology Inc., Santa Cruz, CA, USA), 1 : 5000 Akt (Cell Signaling Technology Inc.) and 1 : 3500 Stat3 (Cell Signaling Technology Inc.) containing 1% non-fat dried milk with 0.1% Tween 20 overnight at 4°C. The membrane was washed in TBS containing 0.05% Tween 20, and then incubated for 2 h at room temperature with horseradish peroxidase-conjugated goat anti-rabbit IgG (Southern Biotechnology Associates Inc., Birmingham, AL, USA) diluted 1 : 10 000 in TBS containing 1% non-fat dried milk containing 0.1% Tween 20. The antigen–antibody peroxidase complex was finally detected by enhanced chemiluminescence (Pierce, Rockford, IL, USA) and visualised by exposure to Amersham Hyperfilm (Amersham Life Sciences, Arlington Heights, IL, USA).

## Results

### Localisation of KORs in NSCLC cells

**Figure 1:**
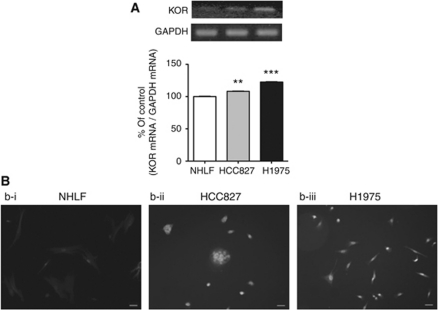
Expression of *κ*-opioid receptors in either NHLF, HCC827 or H1975 cells. (**A**) Upper: Representative RT-PCR for mRNAs of *κ*-opioid receptors and GAPDH, an internal standard, in each cell type. Lower: The intensity of the bands was determined semiquantitatively using ImageJ (National Institute of Health, Bethesda, MD, USA). The values for *κ*-opioid receptor mRNA were normalised by the value for GAPDH mRNA. Data represent the mean with s.e.m. of five independent samples (^**^P<0.01, ^***^P<0.001 *vs* NHLF). (**B**) Distribution of the *κ*-opioid receptor-like immunoreactivity in either NHLF (**B**–i), HCC827 (**B**-ii) or H1975 (**B**-iii) cells. Scale bars=50 *μ*m for all panels.

KORs were found in gefitinib-sensitive HCC827 cells, gefitinib-resistant H1975 cells and NHLF cells, as detected by RT-PCR (Figure 1A) and immunoreactivity towards KOR antibody (Figure 1B). The expression of KOR mRNA was significantly increased in HCC827 cells (*P*<0.01 *vs* NHLF) and H1975 cells (*P*<0.001 *vs* NHLF) compared with NHLF (Figure 1).

### Effect of KOR agonist on the growth of the EGFR exon 19 mutant NSCLC cell line HCC827

**Figure 2:**
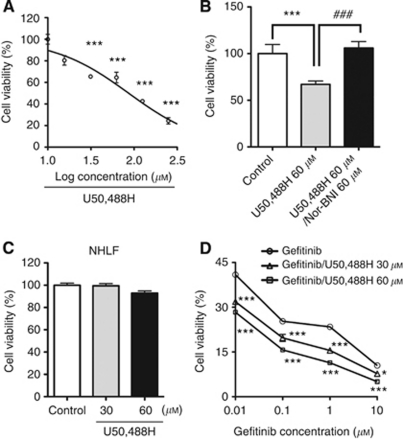
Effect of a *κ*-opioid receptor agonist on the growth of HCC827 cells. (**A**) Suppression of H1975 cell growth by addition of the *κ*-opioid receptor agonist U50,488H (15.6–250 *μ*M) for 2 days. Data represent the mean with s.e.m. of ten independent samples (^***^P<0.001 *vs* non-treated group). (**B**) The suppression of tumour cells by U50,488H was abolished by co-treatment with 60 *μ*M of the *κ*-opioid receptor antagonist nor-BNI. Data represent the mean with s.e.m. of five independent samples (^***^P<0.001 *vs* non-treated group, ^###^*P*<0.001 *vs* U50,488H-treated group). (**C**) Treatment with U50,488H for 2 days had no effect on the growth of NHLF cells. Data represent the mean with s.e.m. of five independent samples. (**D**) Effect of co-treatment with U50,488H and gefitinib on the viability HCC827 cell. The data represent the mean with s.e.m. of five independent samples (F_(3,12)_=12.67, *P*<0.001, gefitinib-treated cells *vs* gefitinib plus U50,488H (30 *μ*M)-treated cells; F_(3,15)_=29.43, *P*<0.001, gefitinib-treated cells *vs* gefitinib plus U50,488H (60 *μ*M)-treated cells; ^*^P<0.05, ^***^P<0.001 *vs* non-treated group).

Addition of the KOR agonist U50,488H to HCC827 cells for 2 days produced a concentration-dependent decrease in tumour cell growth (Figure 2A, *P*<0.001 *vs* non-treated group). This effect was abolished by co-treatment with the selective KOR antagonist nor-BNI (Figure 2B, ^***^P<0.001 *vs* non-treated group, ^###^*P*<0.001 *vs* U50,488H-treated group). In contrast, treatment of NHLF cells with U50,488H did not affect their growth (Figure 2C). In experiments that compared the inhibition of cell growth in cells treated with gefitinib and cells treated with a combination of gefitinib and U50,488H, the growth-inhibitory effects in HCC827 cells were further enhanced in a dose-dependent manner (Figure 2D, *P*<0.001 *vs* gefitinib-treated cells).

### Changes in the growth of gefitinib-resistant H1975 cells by treatment with KOR agonist

**Figure 3:**
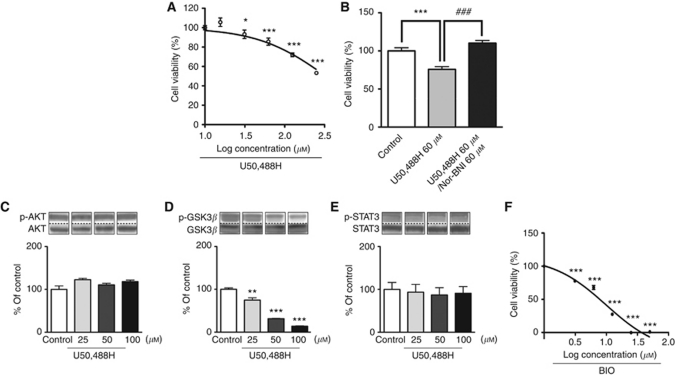
Effect of a *κ*-opioid receptor agonist on the growth of H1975 cells. (**A**) Suppression of H1975 cell growth by the addition of U50,488H (15.6–250 *μ*M) for 2 days. Data represent the mean with s.e.m. of ten independent samples (^*^P<0.05, ^***^P<0.001 *vs* non-treated group). (**B**) U50,488H-induced suppression of tumour cell growth was abolished by co-treatment with 60 *μ*M of the *κ*-opioid receptor antagonist nor-BNI. Data represent the mean with s.e.m. of ten independent samples (^***^P<0.001 *vs* non-treated group, ^###^*P*<0.001 *vs* U50,488H-treated group). Changes in protein levels of p-AKT, p-GSK3*β* and p-STAT3 by treatment of H1975 cells with U50,488H. (**C**–**E**) Cells were treated with U50,488H (25–100 *μ*M) for 2 days. Upper: Representative western blots of p-AKT, p-GSK3*β* and p-STAT3 Lower: Representative western blots of AKT, GSK3*β* and STAT3 in membranous and cytosolic fractions of H1975 cells treated with U50,488H. Each column represents the mean with s.e.m. of five independent samples (^**^P<0.01, ^***^P<0.001 *vs* non-treated group). (**F**) Suppression of the growth of H1975 cells by addition of the selective GSK3*β* inhibitor BIO (3–50 *μ*M) for 2 days. Data represent the mean with s.e.m. of ten independent samples (^***^P<0.001 *vs* non-treated group).

Treatment of gefitinib-resistant H1975 cells with U50,488H for 2 days produced a concentration-dependent and dramatic decrease in tumour cell growth (Figure 3A, *P*<0.001 *vs* non-treated group). This effect was blocked by co-treatment with nor-BNI (Figure 3B, ^***^P<0.001 *vs* non-treated group, ^###^*P*<0.001 *vs* U50,488H-treated group).

### Effect of KOR agonist on the levels of phosphorylated Akt, GSK3*β* and Stat3 in H1975 cells

There were no changes in the levels of either p-Akt or p-Stat3 in H1975 cells by treatment with U50,488H for 2 days (Figures 3C and E). However, the addition of U50,488H to H1975 cells produced a significant and concentration-dependent decrease in p-GSK3*β* (Figure 3D, *P*<0.001 *vs* non-treated group). Furthermore, treatment with a specific GSK-3*β* inhibitor BIO produced a concentration-dependent and significant decrease in tumour cell growth (Figure 3F, *P*<0.001 *vs* non-treated group).

## Discussion

In the present study, we investigated the role of KOR in NSCLC cells using gefitinib-sensitive HCC827 and gefitinib-resistant H1975 cells. We found that KORs were highly expressed in both cell lines. Under these conditions, addition of the selective KOR agonist U50,488H to either HCC827 or H1975 cells produced a concentration-dependent decrease in tumour cell growth. Although some of the doses of U50,488H were relatively high, these effects were abolished by co-treatment with the selective KOR antagonist nor-BNI. These results support the idea that U50,488H can pharmacologically act on KORs to decrease tumour growth. Additionally, the inhibition of tumour growth by gefitinib in HCC827 cells was further enhanced by co-treatment with U50,488H. These findings suggest that the stimulation of KOR may provide unique opportunities for the prevention and treatment of NSCLC.

GSK3*β* is a multifunctional serine/threonine kinase that phosphorylates and thereby regulates the functions of many metabolic, signaling, and structural proteins and transcriptional factors (Grimes and Jope, 2001). EGF can inactivate GSK3*β*, leading to the degradation of c-Myc and *β*-catenin, which are overexpressed in tumour cells. Furthermore, the tumour suppressor p53 can be inactivated because of inactive GSK3*β*. It has been reported that the progressive inactivation of GSK3*β*, which is related to the increase in phosphorylation of GSK3*β*, is critical for the progression of lung cancer (Tian *et al*, 2006). In this study, treatment of H1975 cells with U50,488H produced a significant decrease in the phosphorylation of GSK3*β*. It has been recognised that activated protein kinase A (PKA) leads to phosphorylation of GSK3*β* (Fang *et al*, 2000), whereas activated JNK increases GSK3*β* activity (Hu *et al*, 2009). It should be noted that the stimulation of KOR suppresses cAMP production through Gi proteins, which leads to the inactivation of PKA (Tso and Wong, 2003). Furthermore, the stimulation of KOR invokes the JNK cascade (Kam *et al*, 2004). Although the exact mechanism of KOR-mediated GSK3*β* activation remains unclear at this time, we propose that the stimulation of KOR may activate GSK3*β* through inhibition of the cAMP/PKA pathway and/or activation of the JNK pathway in NSCLC, resulting in the prevention of cancer.

In conclusion, the present results suggest that stimulation of KOR reduces the growth of NSCLC cells through the activation of GSK3*β*. Furthermore, KOR agonist might be a valuable candidate for preventing gefitinib-resistant NSCLC.
