# Supplementary material for: Effect of κ-opioid receptor agonist on the growth of non-small cell lung cancer (NSCLC) cells
Source: Br J Cancer. 2012 Feb 16;106(6):1148–52. doi: 10.1038/bjc.2011.574 (PMC3304401; doi:10.1038/bjc.2011.574)
Supplement: Supplementary Methods [file bjc2011574x1.doc]

**Supplementary method**

**Immunohistochemistry**

Cells were fixed with 4% paraformaldehyde for 20 min at room temperature and processed for immunohistochemistry. The samples were rinsed twice with PBS and pre-treated with PBS containing 0.3% Triton-X100 for 5 min at room temperature. After being blocked in blocking buffer (PBS containing 5% FBS and 0.3% TritonX) for 1 h at room temperature, the samples were incubated overnight at 4℃ with anti-human KOR-1 (rabbit IgG, 1:500, Santa Cruz Biotechnology, CA, USA). After three washes with PBS, the samples were incubated for 1 h at room temperature with secondary antibodies conjugated with Alexa 546 (Invitrogen, Carlsbad, CA, USA). After being washed with PBS, the samples were mounted on slides and examined with a microscope with a 20 x objective lens (IX 71, Olympus, Co., Tokyo, Japan) and photographed with a digital camera (VB-6000, Keyence, Co., Osaka, Japan).

**RNA preparation and semi-quantitative analysis by reverse transcription-polymerase chain reaction (RT-PCR)**

Total RNA in NHLF, HCC827 and H1975 was extracted using the SV Total RNA Isolation system (Promega, Madison, WI) following the manufacturer's instructions. Purified total RNA was quantified spectrophotometrically at A260. To prepare first-strand cDNA, 1 μg of RNA was incubated in 100 μL of buffer containing 10 mM dithiothreitol, 2.5 mM MgCl_2_, dNTP mixture, 200 U of reverse transcriptase II (Invitrogen), and 0.1 mM oligo-dT12-18 (Invitrogen). Each gene was amplified in 50 μL of PCR solution containing 0.8 mM MgCl_2_, dNTP mixture, and DNA polymerase with synthesized primers of human KOR (sense : 5’- TGTCATTGAGTGCTCCTTGC -3’, antisense : 5’- GGCGTAGAGAATGGGATTCA -3’). Samples were heated at 95°C for 1 min, 55°C for 2 min, and 72°C for 3 min. The final incubation was at 72°C for 7 min. The mixture was run on 2% agarose gel electrophoresis with the indicated markers and primers for the internal standard glyceraldehyde-3-phosphate dehydrogenase. The agarose gel was stained with ethidium bromide and photographed with UV transillumination. The intensity of the bands was analyzed and semiquantified by computer-assisted densitometry using ImageJ software.

**Sample preparation and loading for Western blotting**

Cells were solubilized with buffer containing 20 mM Tris-HCl (pH7.4), 0.3％(w/v) Triton, 3 mM MgCl_2_, 1 M sucrose, 5 mM α-ME, and 1/1,000 protease inhibitor for 15 min. Cell lysates were centrifuged at 2,350 g for 10 min at 4^o^C and the supernatant was retained as the lysate fraction for Western blotting. An aliquot of sample was diluted with an equal volume of 2 x electrophoresis sample buffer (Protein Gel Loading Dye-2X, Amresco, Solon, OH) containing 2% sodium dodecyl sulfate (SDS) and 10% glycerol with 0.2 M dithiothreitol. Proteins (7 μL/lane) were separated by size on 4-20% SDS-polyacrylamide gradient gel using the buffer system of Laemmli (1970) and transferred to nitrocellulose membranes in Tris-glycine buffer containing 25 mM Tris and 192 mM glycine.

**Statistical Analysis**

The statistical significance of differences between groups was assessed with one-way or two-way ANOVA followed by the Bonferroni/Dunn multiple comparison test. The statistical significance of differences between two groups was assessed with Student’s *t*-test.
